# Supplementary material for: A dynamic and multi-responsive porous flexible metal–organic material
Source: Nat Commun. 2018 Aug 6;9:3080. doi: 10.1038/s41467-018-05503-y (PMC6079025; doi:10.1038/s41467-018-05503-y)
Supplement: Supplementary file 1 — Supplemental Information [file 41467_2018_5503_MOESM1_ESM.pdf]

File Name: Supplementary Information

Description: Supplementary Figures and Supplementary Table

The multi-dynamic nature of X-pcu-1-Zn

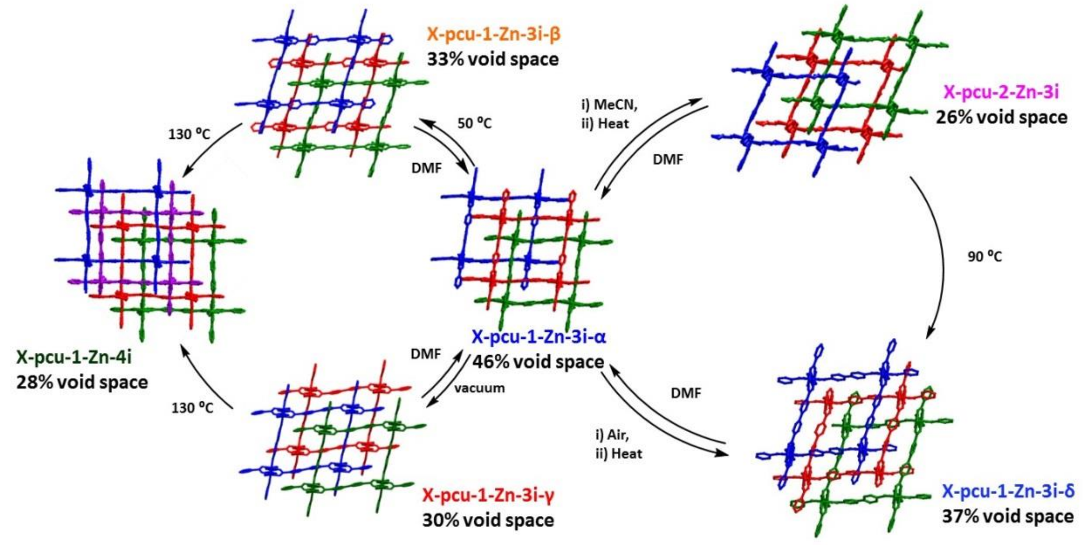

Supplementary Figure 1. Representation of multiple phase transformation under different stimuli. Characterised six phases and transform to four types of structural transformations.

In situ SC-SC X-ray diffraction.

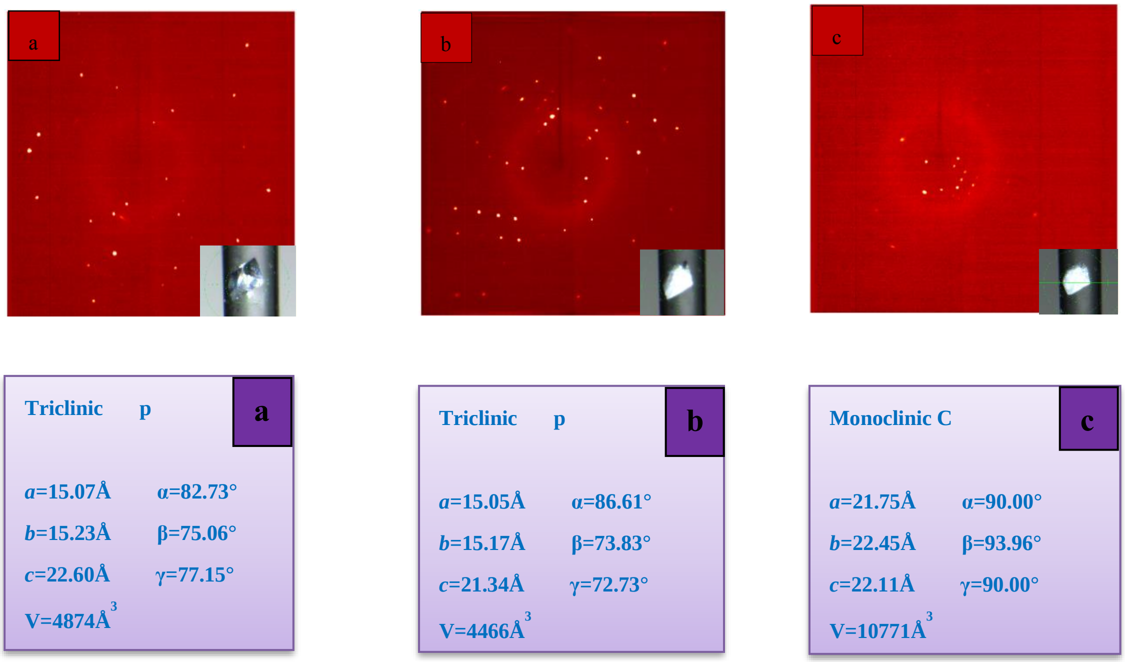

Supplementary Figure 2. _In situ_ variable temperature SC-XRD studies. a) RT, b) 50 °C and c) 130 °C. Transforming to a) X-pcu-1-Zn-3i-α, b) X-pcu-1-Zn-3i-β, and c) X-pcu-1-Zn-4i respectively.

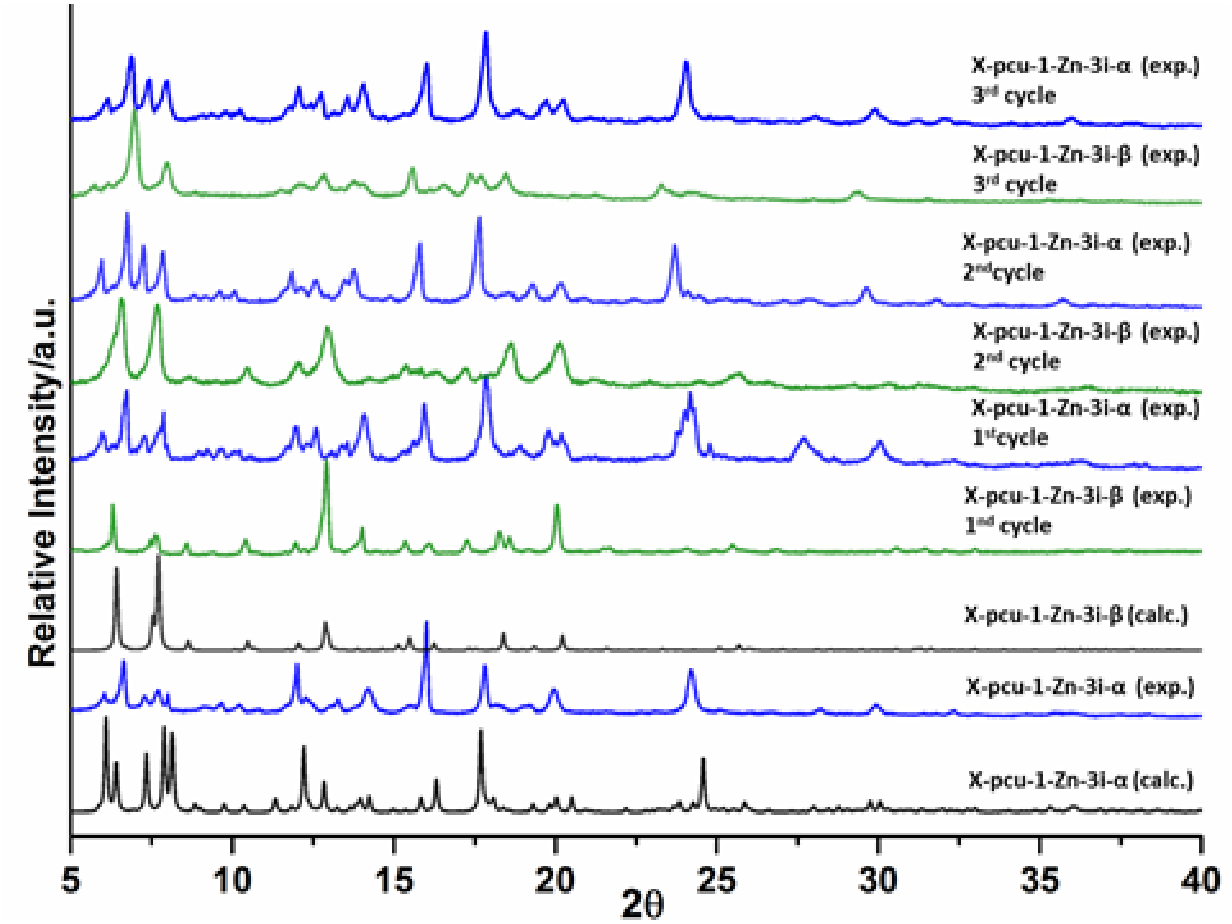

Supplementary Figure 3. Recyclability of X-pcu-1-Zn-3i-β. PXRD pattern of X-pcu-1-Zn-3i-α to X-pcu-1-Zn-3i-β and then X-pcu-1-Zn-3i-β to X-pcu-1-Zn-3i-α three cycles.

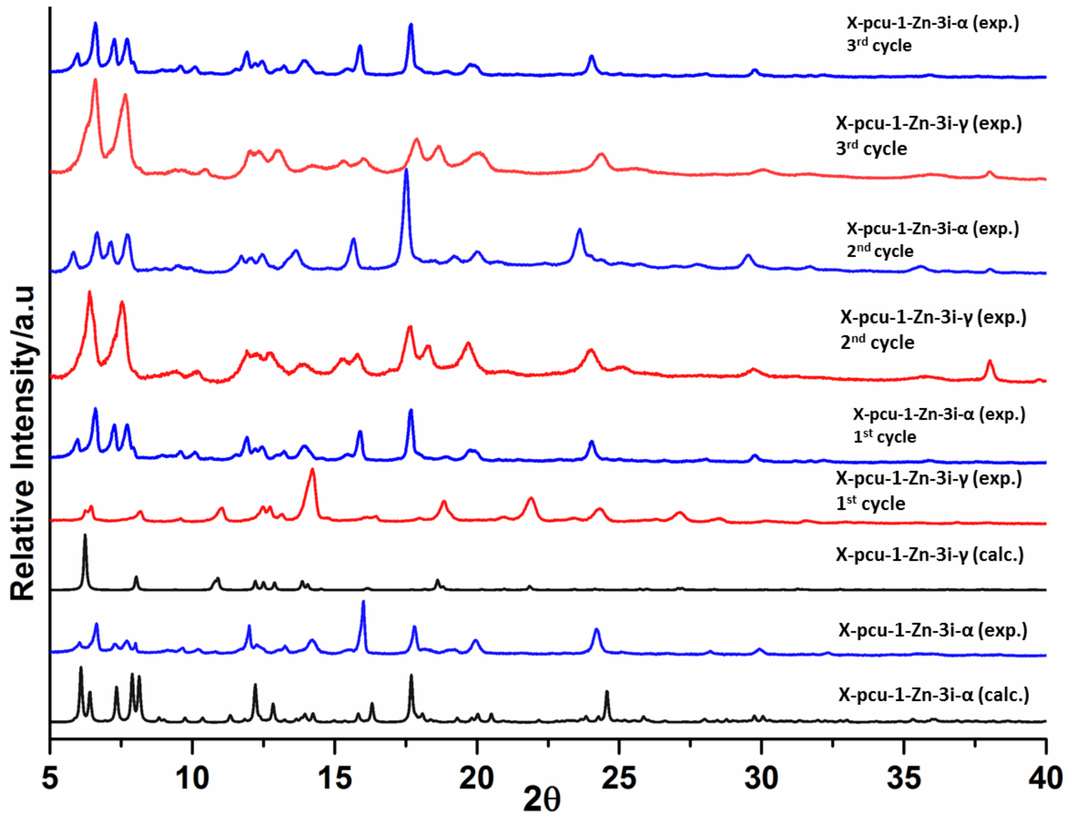

Supplementary Figure 4. Recyclability of X-pcu-1-Zn-3i-γ. PXRD patterns of X-pcu-1-Zn-3i-α to X-pcu-1-Zn-3i-γ and X-pcu-1-Zn-3i-γ to X-pcu-1-Zn-3i-α three cycles.

X-pcu-1-Zn-3i-α

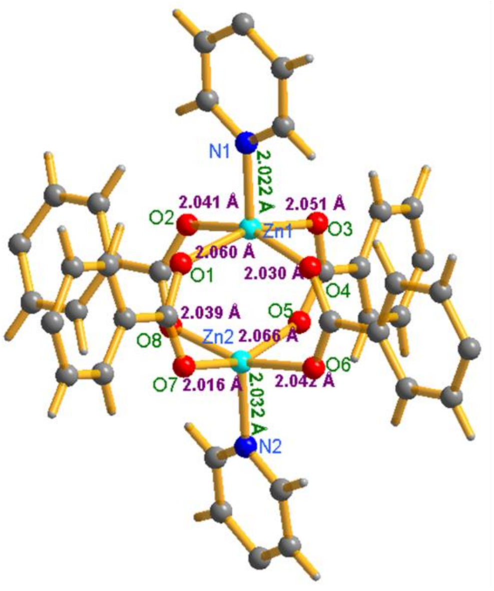

X-pcu-1-Zn-4i

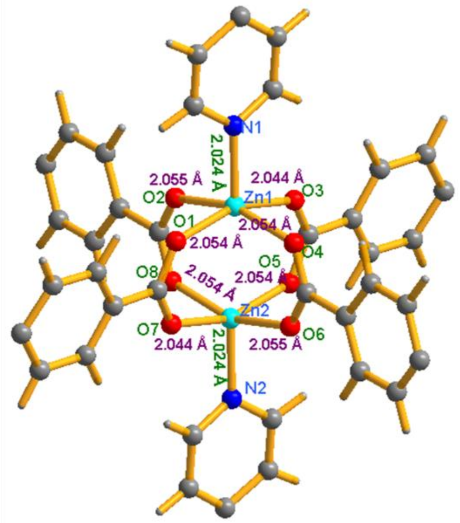

X-pcu-1-Zn-3i-β

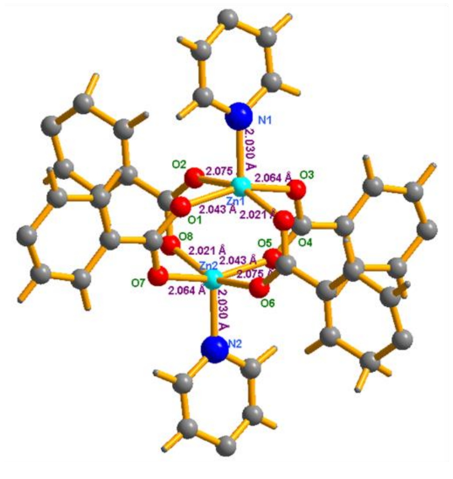

X-pcu-1-Zn-3i-γ

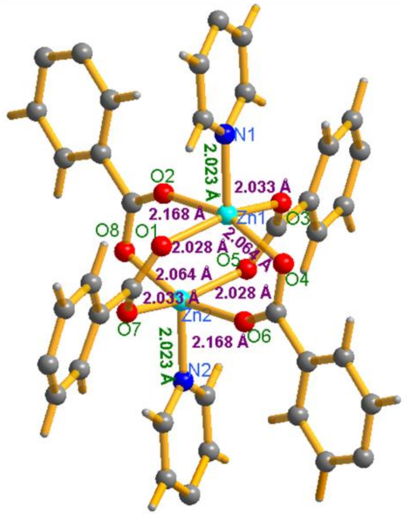

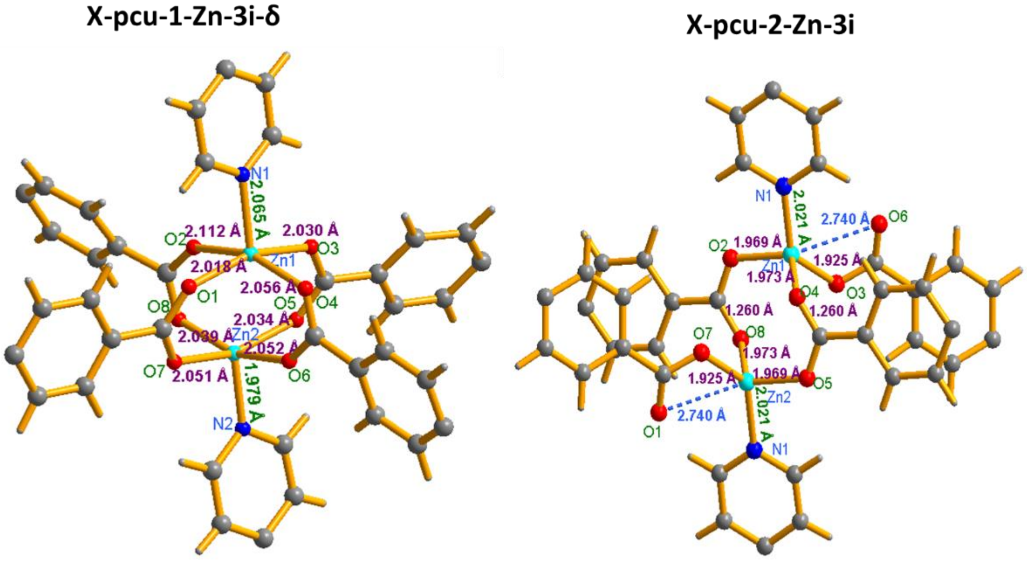

Supplementary Figure 5. Measured bond distances. Showing bond distances between each 4,4′-biphenylene dicarboxylate oxygen with metal and metal to nitrogen distance. Bond distances for Zn–O and Zn–N coordination bonds for each phase.

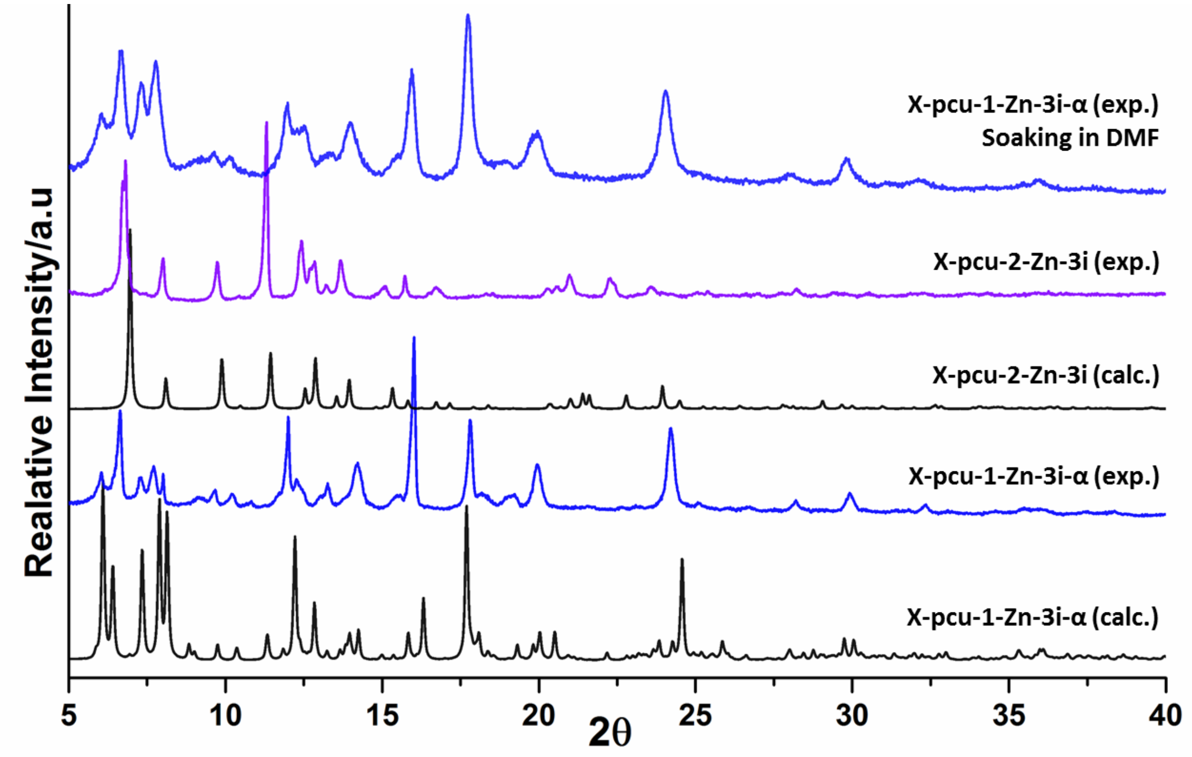

Supplementary Figure 6. Reversibility of X-pcu-2-Zn-3i. PXRD patterns for X-pcu-2-Zn-3i after soaking in DMF for 5 min.

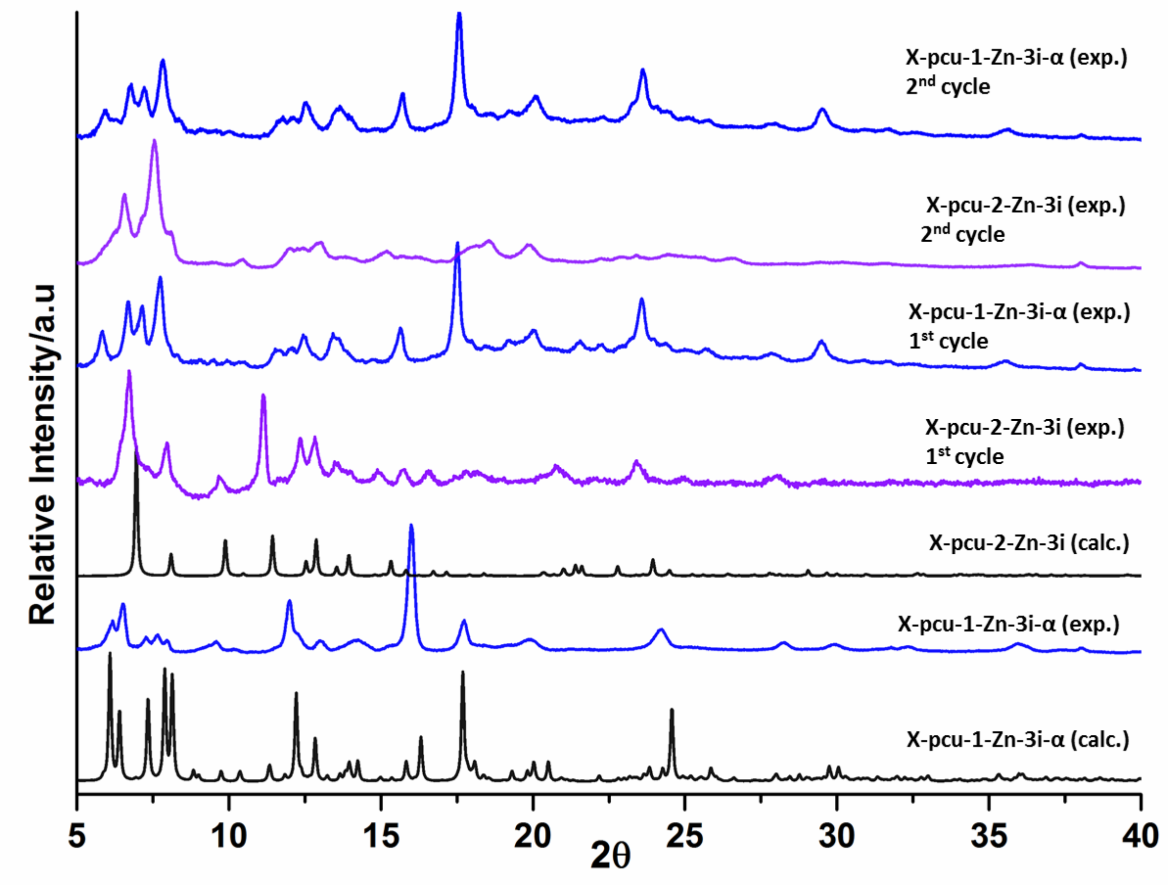

Supplementary Figure 7. Recyclability of X-pcu-2-Zn-3i. PXRD pattern of X-pcu-1-Zn-3i-α to X-pcu-2-Zn-3i and X-pcu-2-Zn-3i to X-pcu-1-Zn-3i-α two cycles.

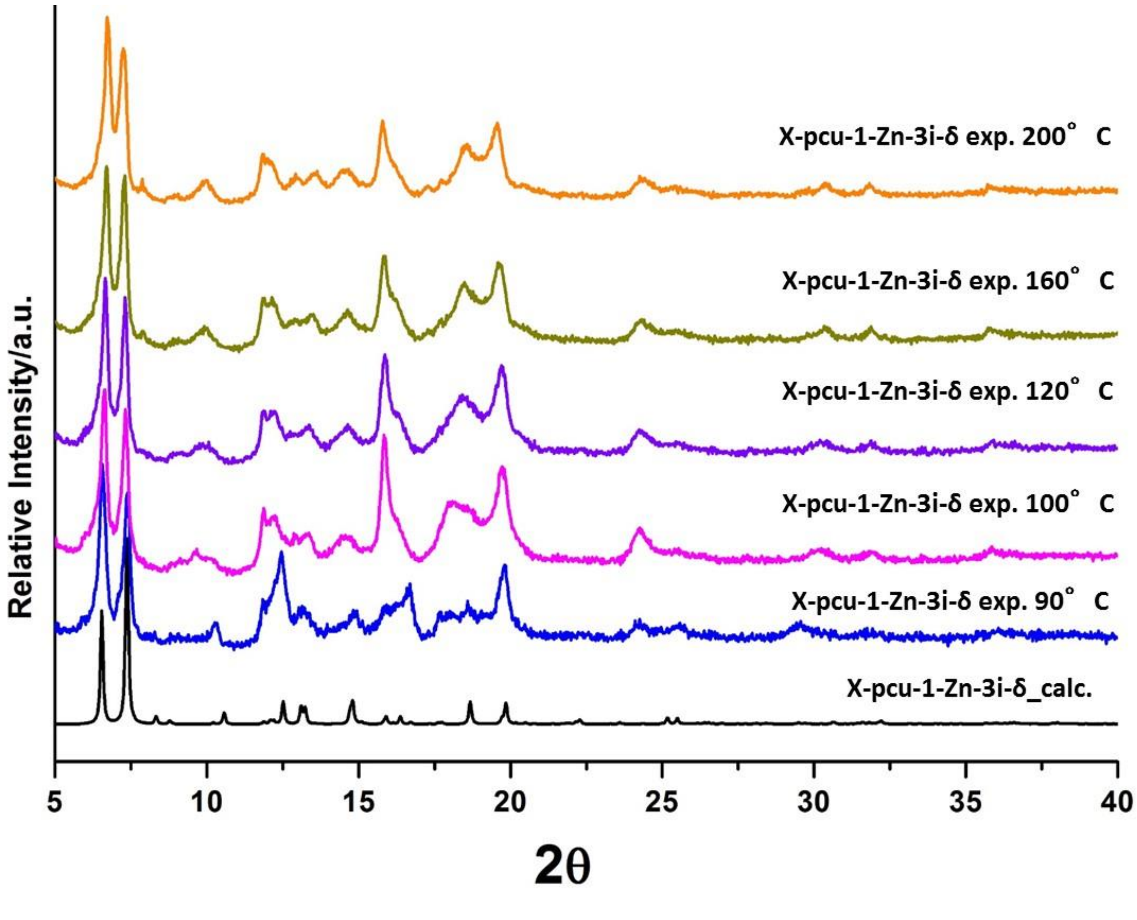

Supplementary Figure 8. Robustness of X-pcu-1-Zn-3i-δ. *In-situ* variable temperature PXRD patterns for X-pcu-1-Zn-3i-δ.

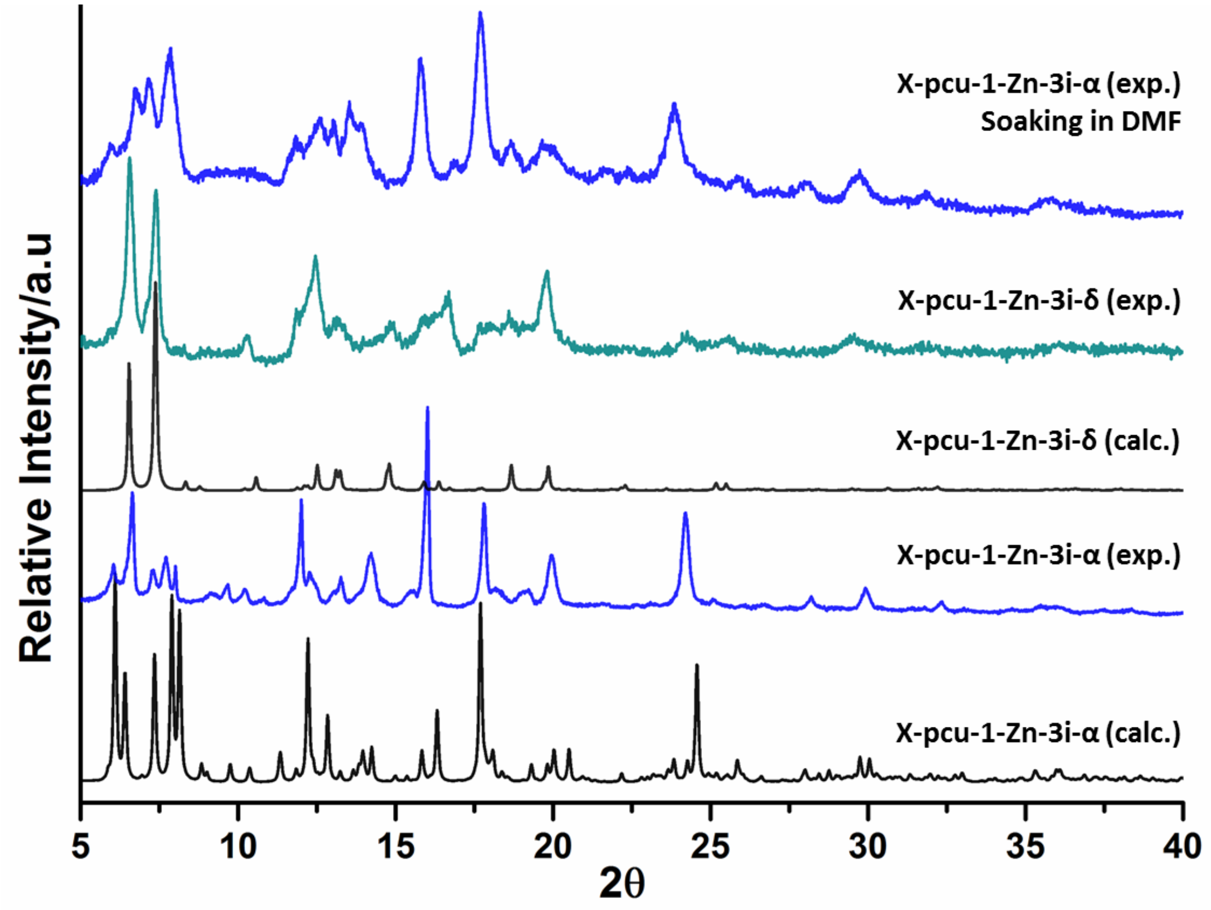

Supplementary Figure 9. Reversibility of X-pcu-2-Zn-3i-δ. PXRD patterns for X-pcu-2-Zn-3i-δ after soaking in DMF for 5 min**.**

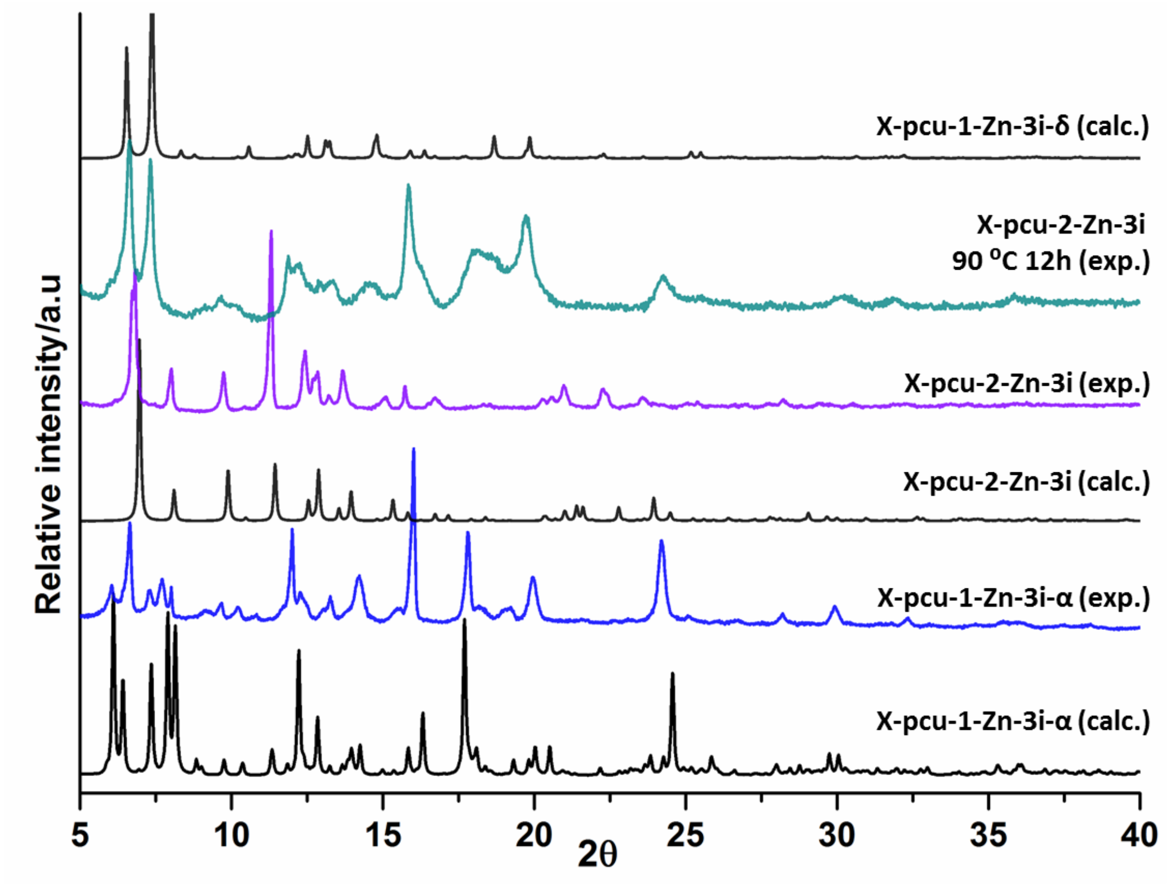

Supplementary Figure 10. Transformation of X-pcu-2-Zn-3i. PXRD comparison of X-pcu-2-Zn-3i after heating at 90 °C for 12 h, leading to conversion into X-pcu-1-Zn-3i-δ.

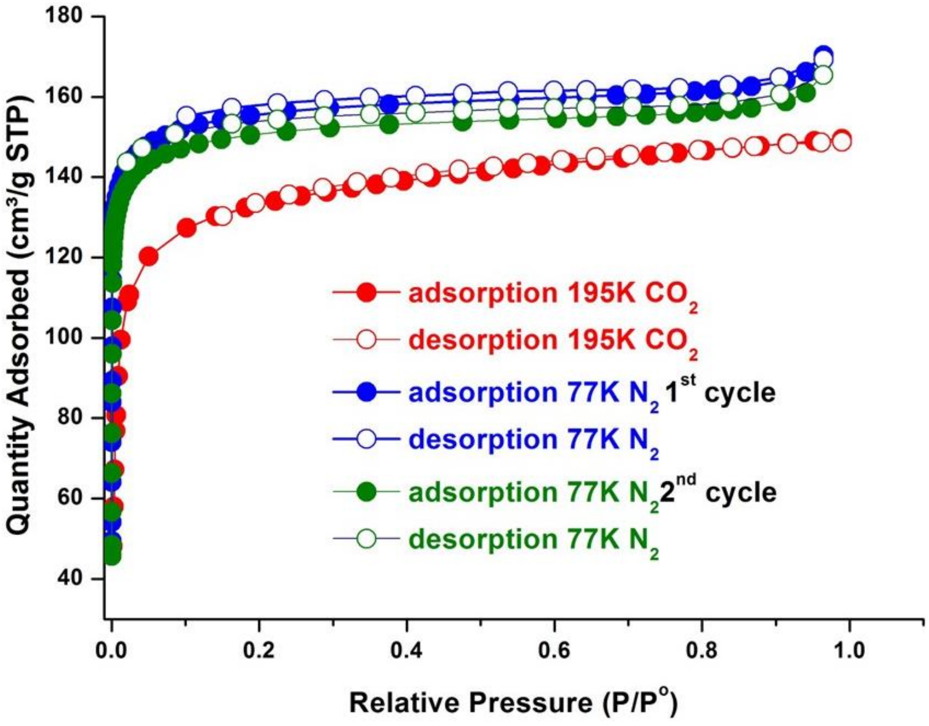

Supplementary Figure 11. Low pressure gas sorption on X-pcu-1-Zn-3i-δ. 195 K CO_2, adsorption (closed red circles) and desorption (open red circles); followed by a 1st cycle of 77 K N_2, adsorption (closed blue circles) and desorption (open blue circles); followed by a 2nd cycle of 77 K N_2, adsorption (closed green circles) and desorption (open green circles).

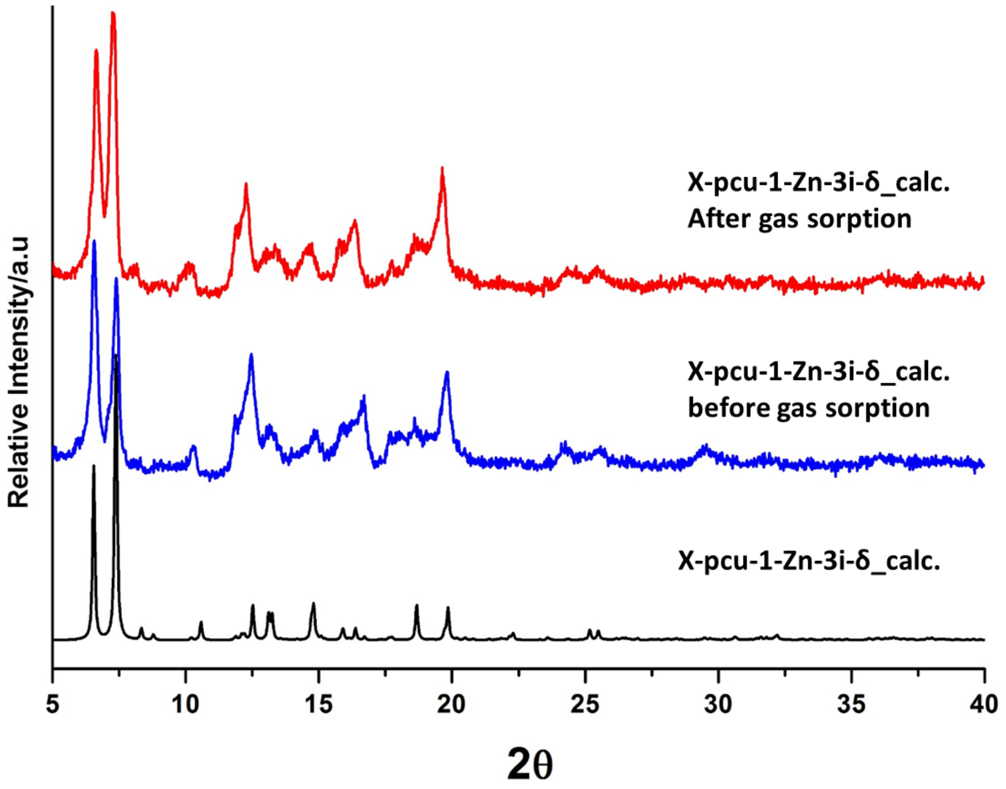

Supplementary Figure 12. Robustness of X-pcu-2-Zn-3i-δ. PXRD patterns for X-pcu-2-Zn-3i-δ after before gas sorption**.**

X-pcu-1-Zn-3i-α

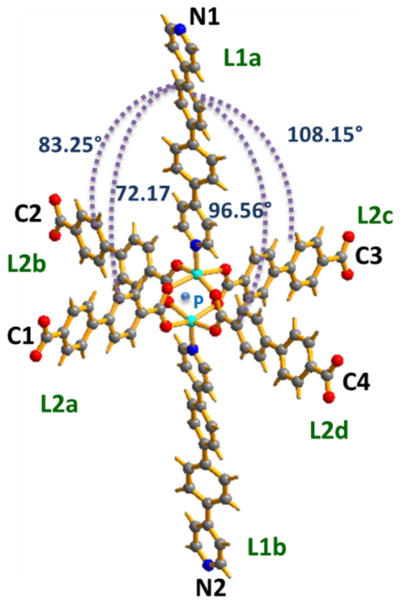

$\angle_{L1a, N1-P-C1, L2a}$ = 72.17°	$\angle_{L1b, N2-P-C1, L2a}$ = 107.66°	$\angle_{L2a, C1-P-C2, L2b}$ = 84.47°
$\angle_{L1a, N1-P-C2, L2b}$ = 83.25°	$\angle_{L1b, N2-P-C2, L2b}$ = 96.67°	$\angle_{L2b, C2-P-C3, L2c}$ = 95.53°
$\angle_{L1a, N1-P-C3, L2c}$ = 108.15	$\angle_{L1b, N2-P-C3, L2c}$ = 72.02°	$\angle_{L2c, C3-P-C4, L2d}$ = 75.48°
$\angle_{L1a, N1-P-C4, L2d}$ = 96.56°	$\angle_{L1b, N2-P-C4, L2d}$ = 83.52°	$\angle_{L2d, C4-P-C1, L2a}$ = 104.52°

X-pcu-1-Zn-3i-β

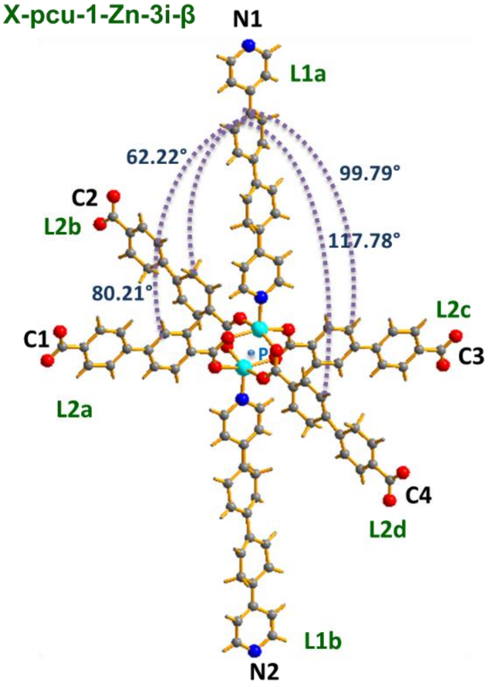

$\angle_{L1a, N1-P-C1, L2a}$ = 80.21°	$\angle_{L1b, N2-P-C1, L2a}$ = 99.17°	$\angle_{L2a, C1-P-C2, L2b}$ = 69.68°
$\angle_{L1a, N1-P-C2, L2b}$ = 62.22°	$\angle_{L1b, N2-P-C2, L2b}$ = 117.29°	$\angle_{L2b, C2-P-C3, L2c}$ = 110.32°
$\angle_{L1a, N1-P-C3, L2c}$ = 99.79°	$\angle_{L1b, N2-P-C3, L2c}$ = 80.21°	$\angle_{L2c, C3-P-C4, L2d}$ = 69.68°
$\angle_{L1a, N1-P-C4, L2d}$ = 117.78°	$\angle_{L1b, N2-P-C4, L2d}$ = 62.18°	$\angle_{L2d, C4-P-C1, L2a}$ = 110.32°

X-pcu-1-Zn-3i-γ

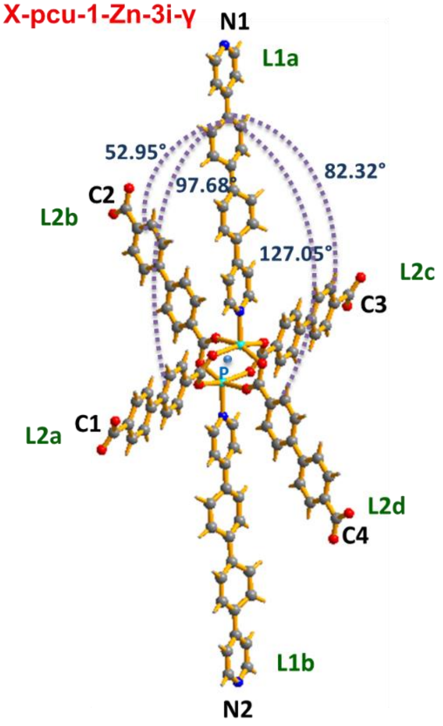

$\angle_{L1a, N1-P-C1, L2a} = 97.68°$	$\angle_{L1b, N2-P-C1, L2a} = 82.32°$	$\angle_{L2a, C1-P-C2, L2b} = 69.03°$
$\angle_{L1a, N1-P-C2, L2b} = 52.95°$	$\angle_{L1b, N2-P-C2, L2b} = 127.05°$	$\angle_{L2b, C2-P-C3, L2c} = 110.97°$
$\angle_{L1a, N1-P-C3, L2c} = 82.32°$	$\angle_{L1b, N2-P-C3, L2c} = 97.68°$	$\angle_{L2c, C3-P-C4, L2d} = 69.03°$
$\angle_{L1a, N1-P-C4, L2d} = 127.05°$	$\angle_{L1b, N2-P-C4, L2d} = 52.95°$	$\angle_{L2d, C4-P-C1, L2a} = 110.97°$

X-pcu-1-Zn-3i-δ

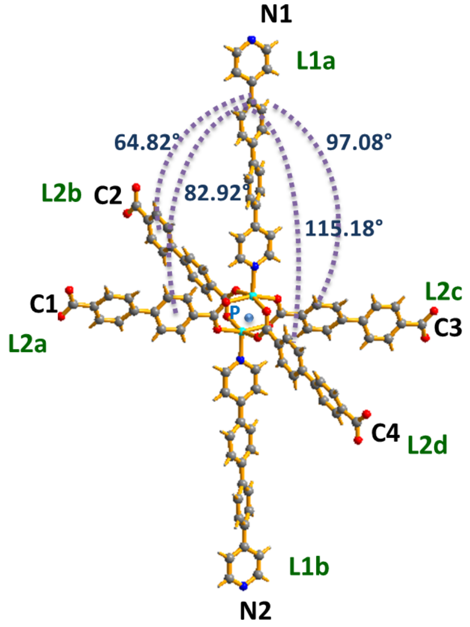

$\angle_{L1a, N1-P-C1, L2a} = 83.10°$	$\angle_{L1b, N2-P-C1, L2a} = 97.03°$	$\angle_{L2a, C1-P-C2, L2b} = 66.56°$
$\angle_{L1a, N1-P-C2, L2b} = 64.78°$	$\angle_{L1b, N2-P-C2, L2b} = 115.21°$	$\angle_{L2b, C2-P-C3, L2c} = 113.87°$
$\angle_{L1a, N1-P-C3, L2c} = 97.01°$	$\angle_{L1b, N2-P-C3, L2c} = 82.86°$	$\angle_{L2c, C3-P-C4, L2d} = 66.49°$
$\angle_{L1a, N1-P-C4, L2d} = 115.27°$	$\angle_{L1b, N2-P-C4, L2d} = 64.73°$	$\angle_{L2d, C4-P-C1, L2a} = 113.03°$

X-pcu-1-Zn-4i

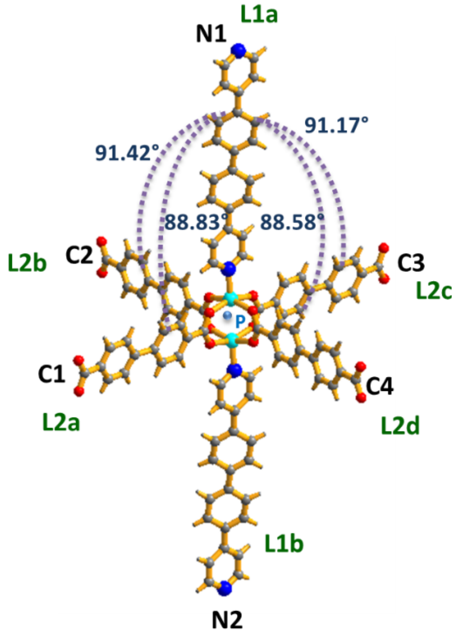

$\angle_{L1a,\ N1\text{-}P\text{-}C1,\ L2a}$ = 88.83°	$\angle_{L1b,\ N2\text{-}P\text{-}C1,\ L2a}$ = 91.42°	$\angle_{L2a,\ C1\text{-}P\text{-}C2,\ L2b}$ = 88.61°
$\angle_{L1a,\ N1\text{-}P\text{-}C2,\ L2b}$ = 91.42°	$\angle_{L1b,\ N2\text{-}P\text{-}C2,\ L2b}$ = 88.83°	$\angle_{L2b,\ C2\text{-}P\text{-}C3,\ L2c}$ = 91.93°
$\angle_{L1a,\ N1\text{-}P\text{-}C3,\ L2c}$ = 91.17°	$\angle_{L1b,\ N2\text{-}P\text{-}C3,\ L2c}$ = 88.58°	$\angle_{L2c,\ C3\text{-}P\text{-}C4,\ L2d}$ = 88.61°
$\angle_{L1a,\ N1\text{-}P\text{-}C4,\ L2d}$ = 88.58°	$\angle_{L1b,\ N2\text{-}P\text{-}C4,\ L2d}$ = 91.17°	$\angle_{L2d,\ C4\text{-}P\text{-}C1,\ L2a}$ = 91.93°

Supplementary Figure 13. Measured bond angles. Between 4,4'-bis(4-pyridyl)biphenyl (L2a), centroid (P) and 4,4′-biphenylene dicarboxylic acid (L1a, L1b, L1c and L1d) for each phase. Angles between each 4,4’-biphenylene dicarboxylic acid with “paddlewheel” centroids (C) are tabulated.

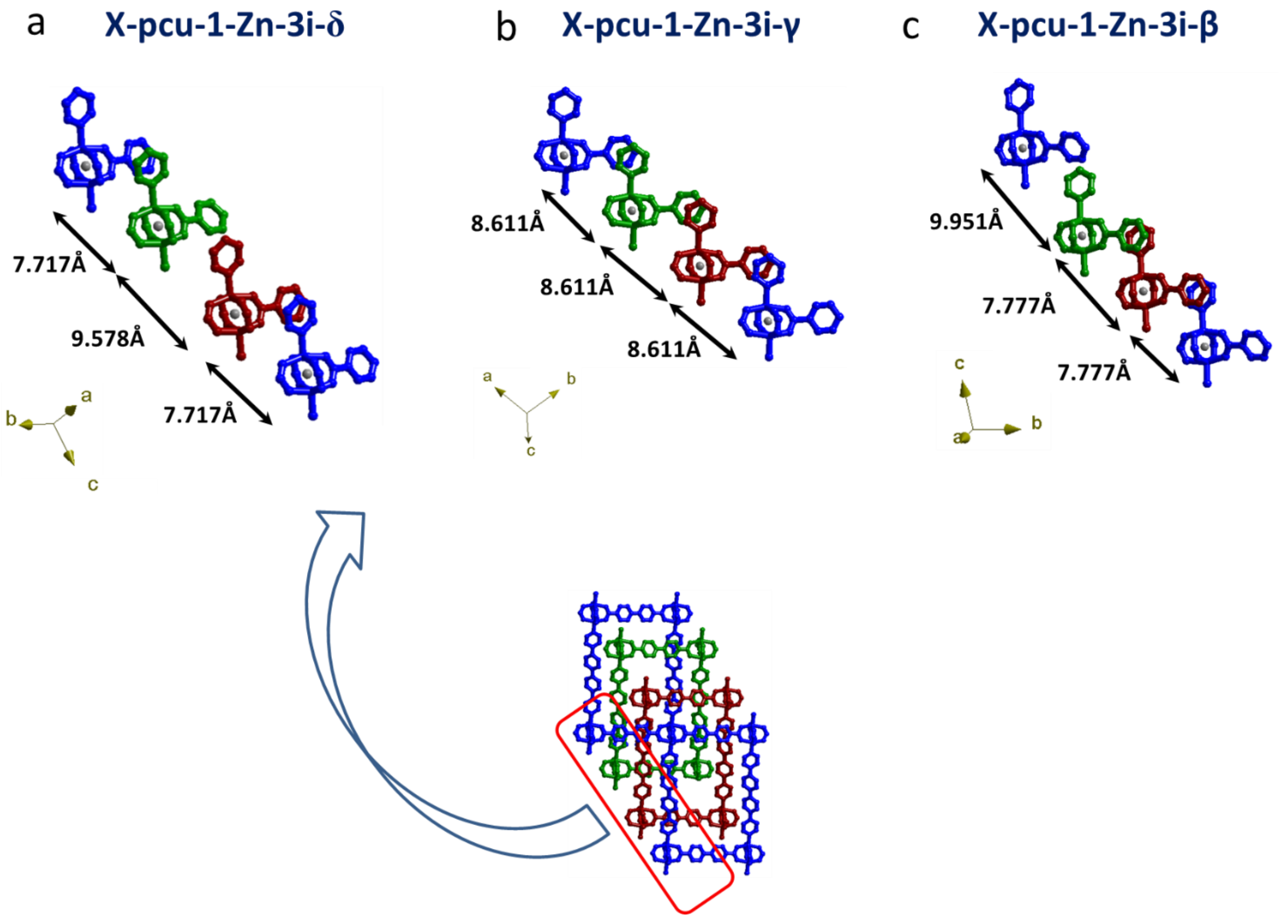

Supplementary Figure 14. **Measured Repeat distances.** Distances measured by considering the centroid of paddlewheel units.in three phases gamma, delta and beta phases.

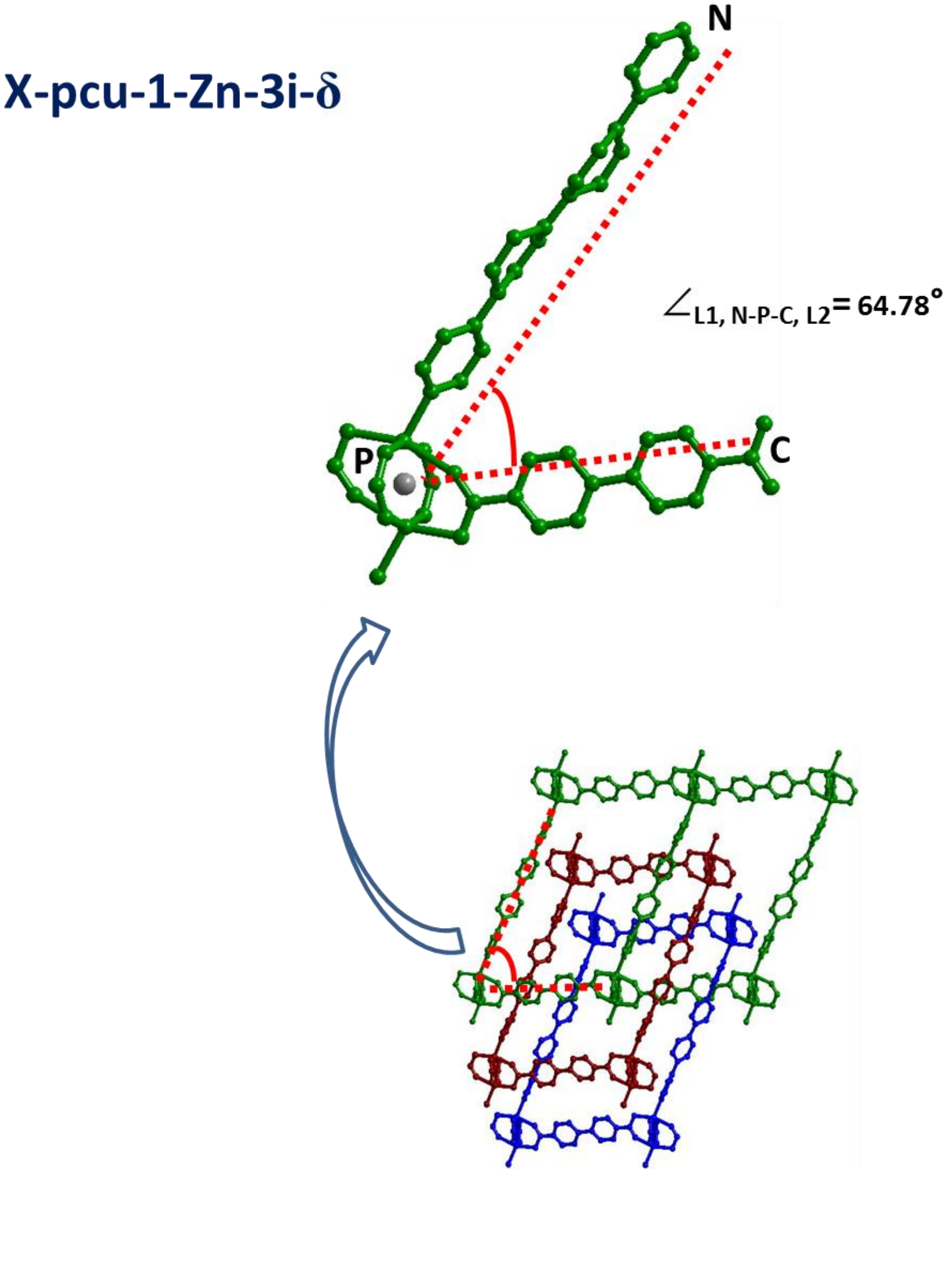

Supplementary Figure 15. **An example for measured bond angle in paddle wheel network.** Angle subtended between N of L1, the centroid (P) of a paddlewheel unit, C, and L2.

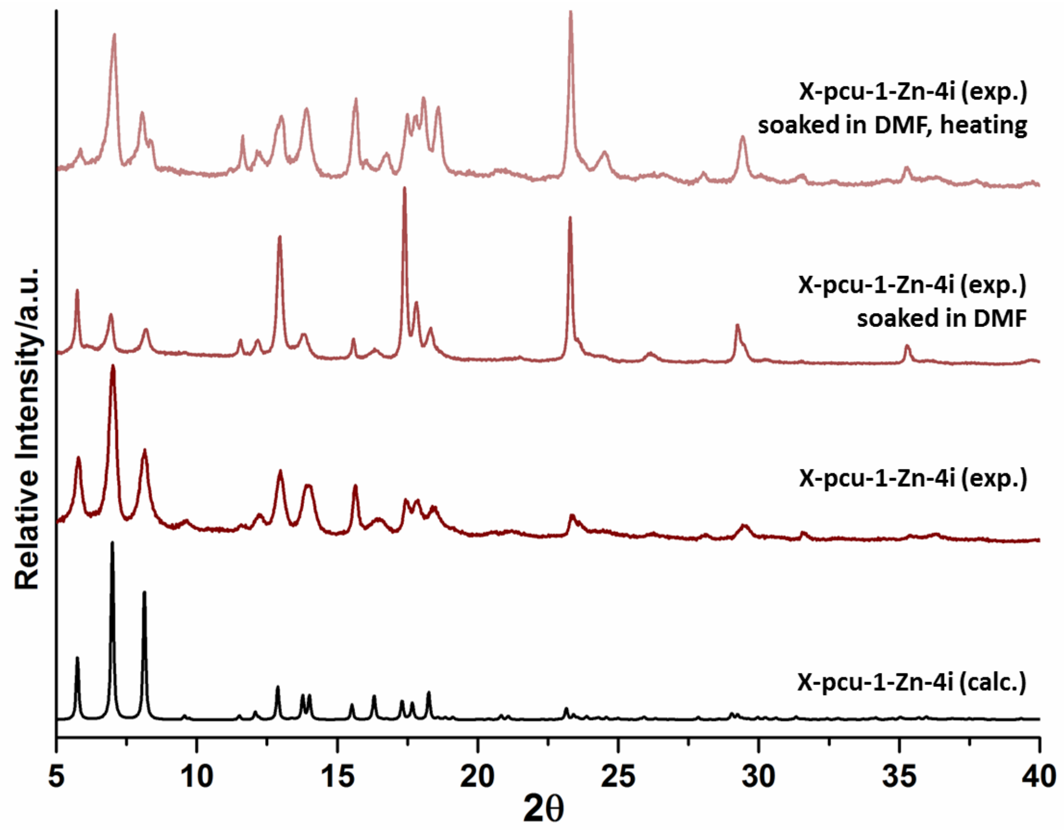

Supplementary Figure 16. Irreversibility of X-pcu-1-Zn-4i. PXRD patterns for X-pcu-1-Zn-4i after soaked in DMF.

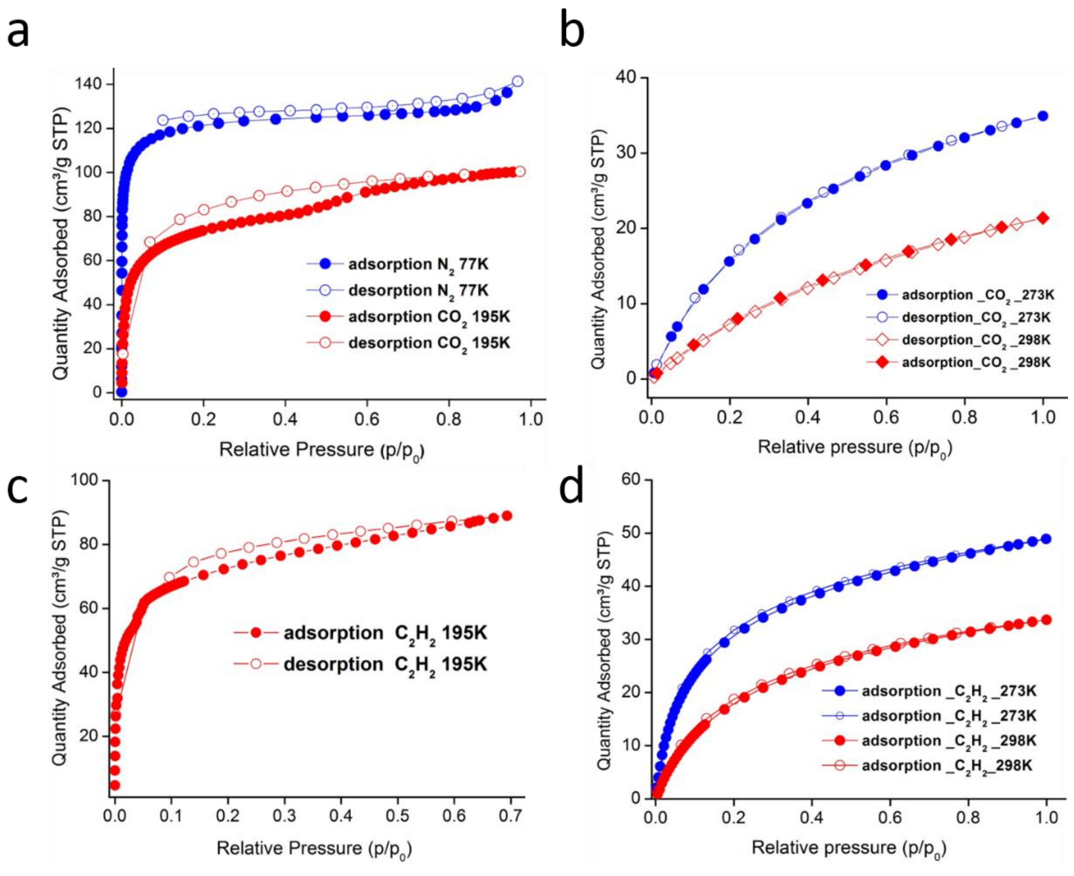

Supplementary Figure 17. Gas sorption on X-pcu-1-Zn-4i. a) 195 K CO_2, adsorption (closed red circles) and desorption (open red circles), 77 K N_2, adsorption (closed blue circles) and desorption (open blue circles). b) Gas sorption, 273 K CO_2, adsorption (closed blue circles) and desorption (open blue circles). 298 K CO_2, adsorption (closed red square) and desorption (open red square). c) Low temperature C_2H_2 adsorption at 195 K (red closed red circles) and C_2H_2 desorption at 195 K (open red circles). d) C_2H_2 adsorption and desorption at 273 K (blue closed and open circles) and C_2H_2 adsorption and desorption at

298 K (red open and closed circles).

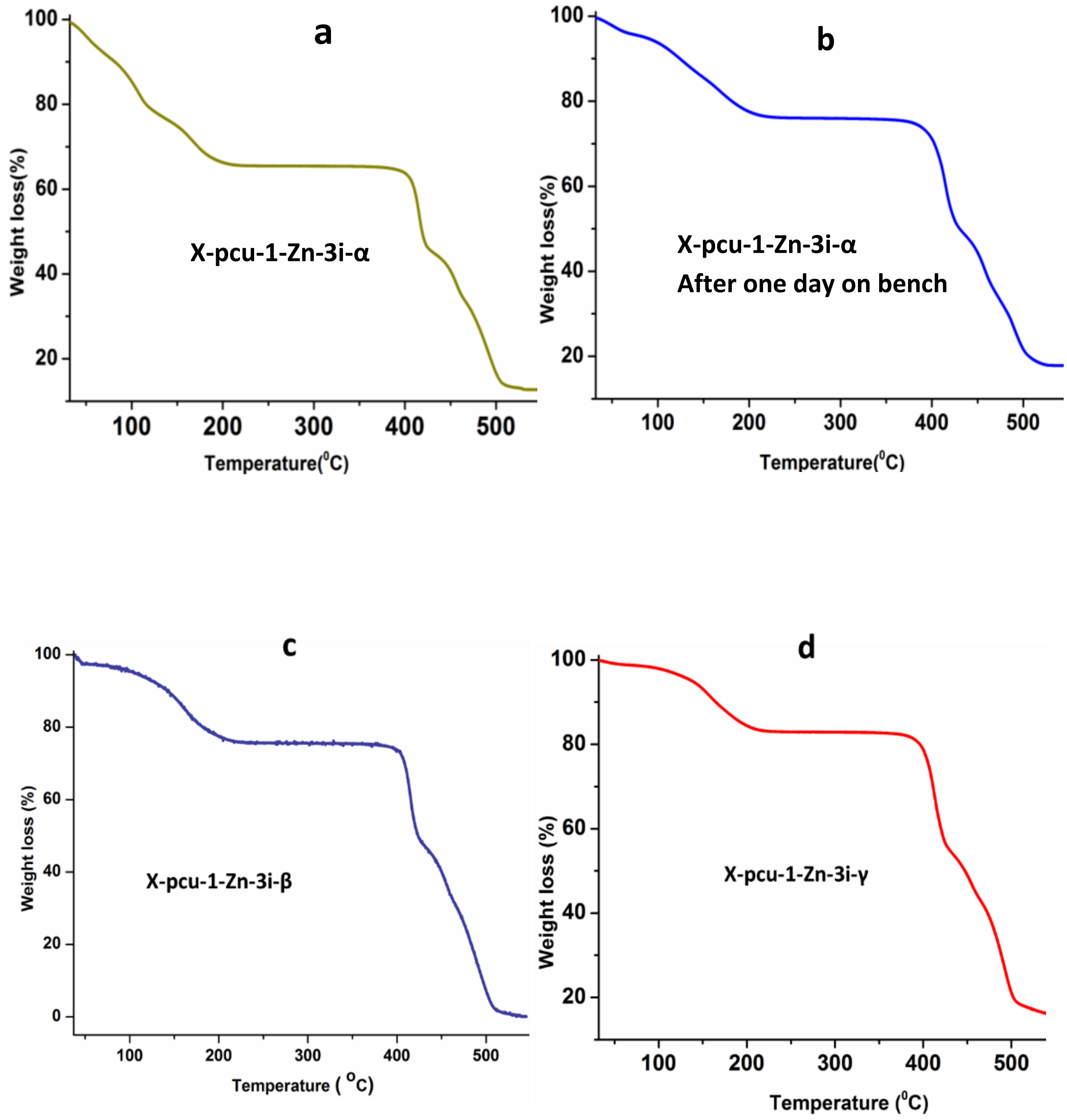

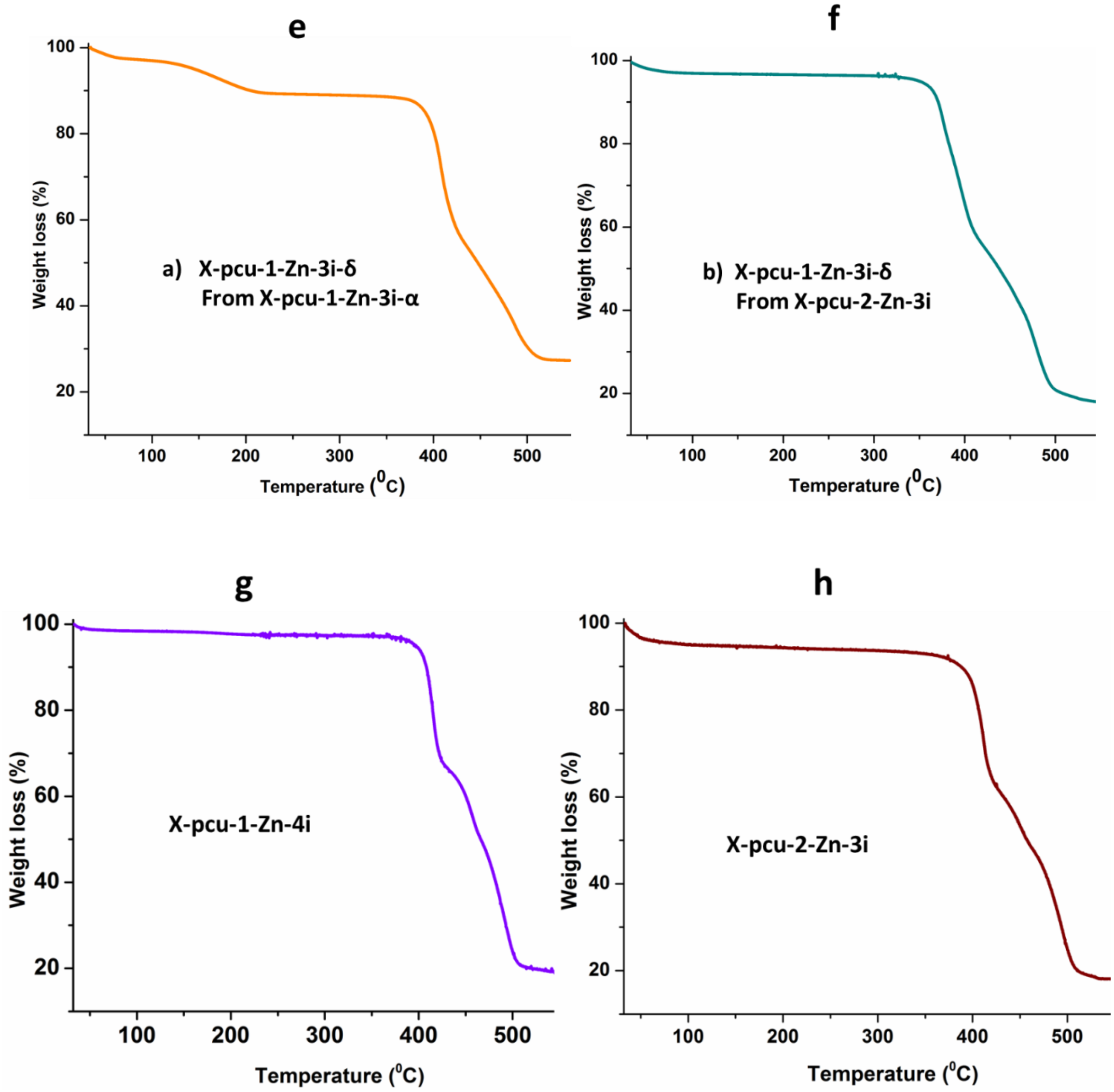

Supplementary Figure 18. Thermo-gravimetric analysis. a) X-pcu-1-Zn-3i-α (bright yellow), weight loss from 40 °C to 200 °C belongs to 35% weight loss, corresponds to ~10 DMF. b) X-pcu-1-Zn-3i-α after one day on bench (blue), 24.6% weight loss, correspond to ~3 H_2O, 5 DMF. c) X-pcu-1-Zn-3i-β (navy blue), 25% weight loss that corresponds to ~6 DMF. d) X-pcu-1-Zn-3i-γ (red), 17% weight loss that corresponds to ~1 DMF. e) X-pcu-1-Zn-3i-δ (orange), 11.5% weight loss corresponds to ~3 H_2O, 2DMF. f) X-pcu-1-Zn-3i-δ (orange), g) X-pcu-1-Zn-4i and h) X-pcu-2-Zn-3i (wine).

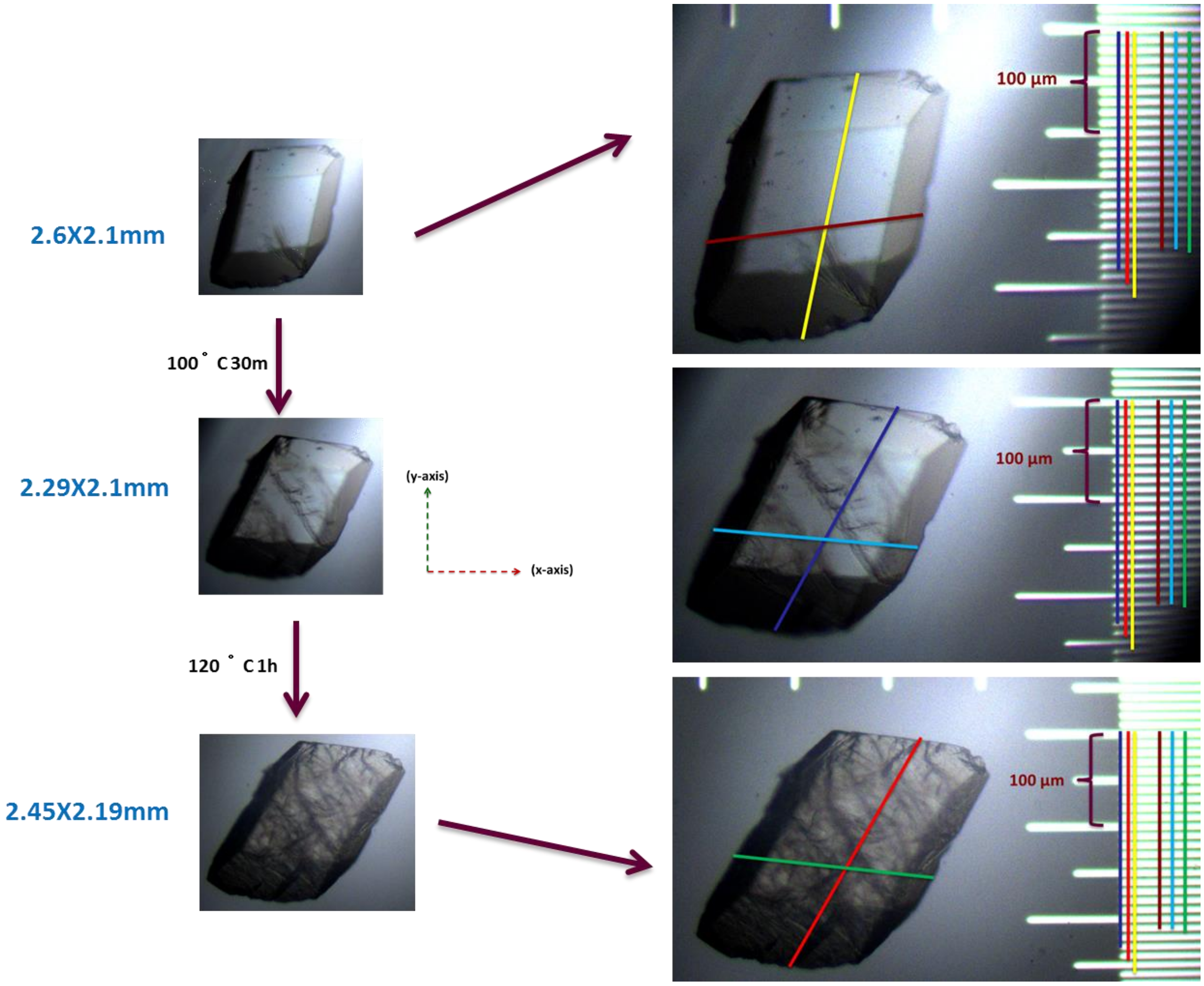

Supplementary Figure 19. SCSC transformation. Decreasing size from single-crystal to single-crystal through change in degree of interpenetration. 12% decreasing size of the crystal along X and Y-axis.

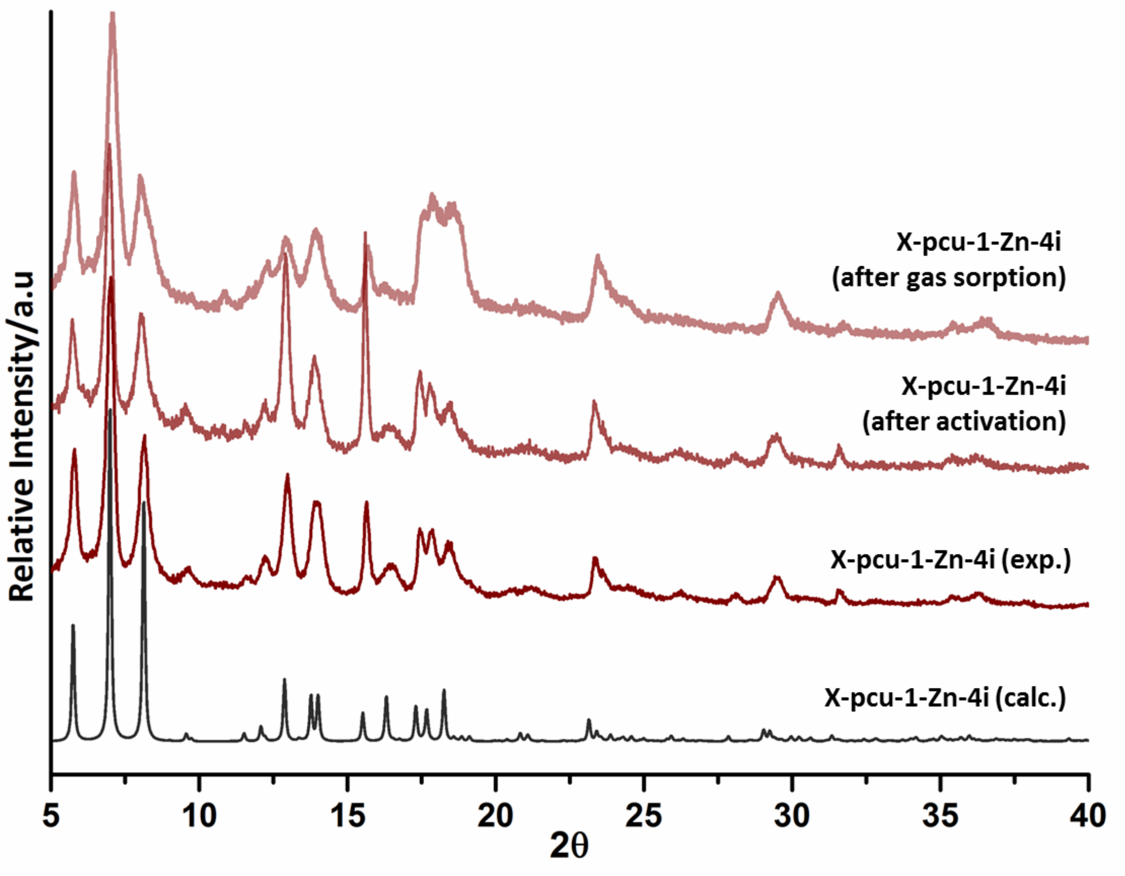

Supplementary Figure 20. Robustness of X-pcu-1-Zn-4i. PXRD patterns for X-pcu-1-Zn-4i, after activated at 130 °C for 12h and after gas sorption**.**

Supplementary Table 1. Crystallographic data and refinement parameters.

	X-pcu-1-Zn-3i-α	X-pcu-1-Zn-3i-α'	X-pcu-1-Zn-3i-β	X-pcu-1-Zn-3i-γ
Formula[a]	$C_{105}H_{118}N_{13}O_{22}Zn_3$	$C_{90}H_{90}N_8O_{20}Zn_3$	$C_{93}H_{90}N_9O_{19}$ Zn_3	$C_{28}H_{23}N_2O_5$ Zn
cF.W.	2110.66	1800.37	1833.33	532.76
T (K)	100(2)	107(2)	301(2)	100(2)
Space group	*P*-1	*P*-1	*P*-1	*P*-1
a (Å)	15.1476(17)	15.0915(7)	15.0289(13)	8.6093(6)
b (Å)	15.2325(18)	15.2091(7)	15.1660(13)	11.2178(9)
c (Å)	22.560(2)	22.5177(10)	21.3049(18)	14.7466(11)
α (°)	84.406(7)	82.213(2)	86.253(5)	95.990(4)
β (°)	74.600(5)	73.867(2)	73.781(4)	104.997(4)
γ (°)	81.271(5)	77.559(2)	71.577(5)	98.069(4)
V (Å^3)	4951.4(10)	4832.5(4)	4422.4(7)	1347.24(18)
Z	2	2	2	2
D_c (g cm^{-3})[a]	1.415	1.090	1.377	1.313
μ (mm^{-1})	1.417	1.343	1.348	1.475
Data collected/unique	40124/14077	13165/7466	55556/ 14060	11162/3794
R_1 (>2σ)	0.0955	0.0921	0.0700	0.0717
wR_2 (>2σ)	0.2689	0.2974	0.1765	0.1779
GOF	1.025	1.104	1.042	1.114

	X-pcu-1-Zn-3i-δ	X-pcu-2-Zn-3i	X-pcu-1-Zn-4i
Formula[a]	$C_{81}H_{68}N_5O_{17}Zn_3$	$C_{25}H_{16}NO_4$ Zn	$C_{25}H_{16}NO_4$ Zn
F.W.	1561.27	623.96	459.76
T (K)	100(2)	100(2)	100(2)
Space group	*P*-1	*P*-1	*C*2/c
a (Å)	15.0486(7)	9.1059(10)	21.279(2)
b (Å)	15.1494(7)	11.0832(12)	21.805(2)
c (Å)	22.0093(10)	13.3354(15)	22.650(2)
α (°)	82.029(2)	79.197(7)	90.00
β (°)	71.942(2)	72.630(7)	91.481(5)
γ (°)	69.186(2)	88.477(7)	90.00
V (Å^3)	4456.8(4))	1261.08(2)	10505.6(17)
Z	2	2	16
D_c (g cm^{-3})[a]	1.136	1.642	1.163
μ (mm^{-1})	1.338	1.576	1.513
Data collected/unique	38319/13523	12090/3830	30537/7196
R_1 (>2σ)	0.0774	0.0438	0.1876
wR_2 (>2σ)	0.2235	0.1011	0.3984
GOF	1.111	1.033	1.057

[a] For phases α, β, γ, δ modelling of solvent present in structural pores was either not possible, or possible only partially due to the disorder; type and amount of solvent present in the structure was determined based on TGA analysis and Formulas in this table were updated accordingly. Similarly, values for *Dc* were calculated based on the actual weight of components present in the crystal, evidenced in TGA. Due to the disorder of DMF molecules present in X-pcu-1-Zn-3i-α their accurate refinement was hindered. As a result only 7 out of 10 DMF molecules present in the asymmetric cell were found and refined. Additionally, based on the residual electron density distribution, as well as size and shape of the thermal ellipsoids of atoms in DMF molecules, it can be deduced that DMF molecules are disordered over multiple positions in proximity to the assigned sites. This disorder could not be modelled properly due to its complexity.
